# The Experiences of Midwives in Caring for Vulnerable Pregnant Women in The Netherlands: A Qualitative Cross-Sectional Study

**DOI:** 10.3390/healthcare11010130

**Published:** 2022-12-31

**Authors:** Evi Vlassak, Kathelijne Bessems, Jessica Gubbels

**Affiliations:** Department of Health Promotion, NUTRIM School of Nutrition and Translational Research in Metabolism, Maastricht University, P.O. Box 616, 6200 MD Maastricht, The Netherlands

**Keywords:** pregnancy, pregnant women, midwifery, health disparity, minority and vulnerable populations, qualitative research

## Abstract

Vulnerable pregnant women have an increased risk for preterm birth and perinatal mortality. This study identifies the perspectives, perceived barriers, and perceived facilitators of midwives toward current care for vulnerable pregnant women in the Netherlands. Knowing those perspectives, barriers, and facilitators could help increase quality of care, thereby reducing the risks of preterm birth and perinatal mortality. Midwives working in primary care practices throughout the Netherlands were interviewed. Semi-structured interviews were conducted remotely through a video conference program, audio recorded, transcribed verbatim, and coded based on the theoretical domains framework and concepts derived from the interviews, using NVivo-12. All midwives provided psychosocial care for vulnerable pregnant women, expected positive consequences for those women resulting from that care, considered it their task to identify and refer vulnerable women, and intended to improve the situation for mother and child. The main barriers perceived by midwives were too many organizations being involved, inadequate communication between care providers, lack of time to care for vulnerable women, insufficient financing to provide adequate care, and uncooperative clients. The main facilitators were having care coordinators, treatment guidelines, vulnerability detection tools, their own knowledge about local psychosocial organizations, good communication skills, cooperative clients, consultation with colleagues, and good communication between care providers. The findings suggest that midwives are highly motivated to care for vulnerable women and perceive a multitude of facilitators. However, they also perceive various barriers for providing optimal care. A national guideline on how to care for vulnerable women, local overviews of involved organizations, and proactive midwives who ensure connections between the psychosocial and medical domain could help to overcome these barriers, and therefore, maximize effectiveness of the care for vulnerable pregnant women.

## 1. Introduction

Although perinatal mortality in the Netherlands has long been high compared to other European countries [1], a clear decrease started in 2004 [2]. However, since 2018, perinatal mortality in the Netherlands has been on the rise again [3].

Almost two-thirds of perinatal mortality is attributable to preterm birth [3]. Preterm birth and associated perinatal mortality are particularly common in vulnerable pregnant women [4]. Vulnerable women are women who are exposed to physical, psychological, cognitive, and/or social risk factors in combination with lack of adequate support and/or adequate coping skills [5]. Common characteristics of vulnerable pregnant women are a low educational level; lack of sufficient and/or stable income; bad living conditions; social isolation; insufficient knowledge and skills regarding their health; a migration background; a young maternal age; and a lack of knowledge of how to act when care is needed [6,7].

The majority of Dutch pregnant women (86%) start obstetric care in a midwife-led care practice (i.e., primary care) [8]. Therefore, midwives from midwife-led care practices are crucial for improving the care for vulnerable women. One of the core responsibilities of midwives is to identify vulnerable women, because not all vulnerable women might be aware of their own risks, or might not seek help when needed [9]. However, previous research found that instruments available for identifying vulnerability (i.e., vulnerability detection tools), are currently underused by midwives [10]. Extra guidance can reduce the risk of preterm birth [11]. To be able to provide this guidance, vulnerable women should be referred to specialized care providers [11]. However, this referral is often lacking [9]. Additionally, vulnerable women often have poor attendance to maternity care [12], increasing their risk for perinatal mortality [13]. How midwives approach these women could influence women’s attendance [14]. Previous research found that vulnerable women, in maternity care, value being treated as an individual, making informed choices, and feeling safe [15].

The factors related to midwives’ role in identifying, referring, and increasing attendance of vulnerable pregnant women in prenatal care, are poorly understood. No previous studies that assess the role of midwives in identifying, referring, or increasing attendance of vulnerable pregnant women in prenatal care were found. The perspectives of midwives regarding barriers and facilitators of high-quality care for vulnerable pregnant women are therefore currently unknown. Knowing those determinants can help structure and improve current care. The theoretical domains framework (TDF) [16,17] has previously been used to describe barriers and facilitators that midwives experience in promoting healthy behavior among pregnant women in general [18]. The TDF describes 14 domains of determinants of high-quality care, including social influences, skills, and environmental context and resources.

The main objective of this qualitative study is to identify the perspectives, perceived barriers, and perceived facilitators of midwives toward care for vulnerable pregnant women in the Netherlands. This will be done using the TDF domains. The secondary objectives are to explore the three core aspects of the care, namely identifying vulnerable women; referring them to specialized care providers; and increasing their attendance in prenatal care and to form recommendations to improve those core aspects.

## 2. Materials and Methods

### 2.1. Study Design

The current study used a qualitative cross-sectional design.

### 2.2. Participants

Participants were included in the study if they worked in midwifery-led care for at least half a year and cared for at least one vulnerable pregnant woman in the year before the interview. This ensured they had experience in working with vulnerable pregnant women in primary care. Additionally, participants had to speak Dutch or English.

### 2.3. Procedures

Participants were recruited using consecutive and snowball sampling. Maternity care collaborations (MCC) were contacted via email and were requested to share a participation invitation with their connected primary care midwives, as well as on their knowledge sharing platform. MCCs have a central role in maternity care policy development, and stimulate collaboration between midwife-led care practices and regional maternity care professionals [19]. All Dutch midwives are advised to be connected to such MCCs [20]. The MCCs were asked to confirm whether they forwarded the invitation to the midwives within their MCC. If there was no response after two weeks, a reminder was sent. Using this method, 53 of the 71 Dutch MCCs (75%) shared the invitation. Additionally, the participation invitation was posted in the Facebook group of the Royal Dutch Association of Midwives. Furthermore, after each interview, participants were asked to distribute the invitation to midwives in their network. The participation invitation included details regarding the researcher’s background, the relevance and objective of the research, and practical information regarding the interviews. Recruitment ended after data saturation, defined as receiving no new information in two consecutive interviews.

### 2.4. Data Collection

The current study explored midwives’ experiences of current care for vulnerable pregnant women, using semi-structured interviews. This allowed participants to openly discuss personal opinions and feel unrestricted to discuss new concepts. Ethical approval for this study was given by ethics board of Maastricht University. The consolidated criteria for reporting qualitative research (COREQ) [21] were used to ensure all aspects of the qualitative research were reported. The interviews were conducted between April and May 2021 by the first author (EV), a qualified midwife and health promotion MSc. Participants were asked for written informed consent to participate and being audio recorded. Participants verbally confirmed their written consent before the interview. All audio files were deleted after verbatim transcription. Every participant was interviewed once. As a consequence of the COVID-19 pandemic and ensuing regulations limiting in person contact [22], the interviews were conducted remotely through a secured video conference program.

### 2.5. Instruments

An interview guide (Appendix A, Table A1), developed specifically for the current study, was used for all interviews. The interview guide included questions about the background of the participants (i.e., age, experience, location of employment, average work week, connection to MCCs, and education). Additionally, the guide included questions based on each of the 14 TDF domains [16,17]: knowledge, skills, professional role and identity, beliefs about capabilities, optimism, beliefs about consequences, reinforcement, intentions, goals, memory/attention/decision processes, environmental context/resources, social influences, emotion, and behavioral regulation (Table 1). In the interview guide, behaviors of interest were specified as: identifying of vulnerable pregnant women, referral to specialized care, and increasing attendance in prenatal care. No definition of vulnerable pregnant women was given to the participants, as participants were asked to give their own definition of vulnerable women to identify their perspectives. Before the first interview, the interview guide was reviewed for relevance and comprehensibility by an independent qualified midwife. The questions were adjusted accordingly.

### 2.6. Analysis

All transcripts were anonymized. A codebook was developed based on the concepts of the TDF and additional concepts derived from the interviews. Codes were assigned (by EV) to data fragments of the transcript (open coding). Then, the codes were reformed to main- and subcategories (axial coding). Finally, core categories were formed by integrating concepts (selective coding). NVivo-12 was used to facilitate data coding, structuring, and analysis. The codebook (Appendix B, Table A2) contained a total of 15 main nodes, 26 secondary nodes, and 20 tertiary nodes. One randomly selected interview was coded separately by the first author (EV) and a second coder (MSc health promotion). Based on this, a Cohen’s Kappa interrater reliability ratio of 0.90 was calculated, which can be considered strong [23]. To support the presentation of the results, quotes were translated into English (EV). Translations were checked by a second member of the research team (JG).

## 3. Results

### 3.1. Participants and Interviews

All interviews (N = 19) were conducted in Dutch and lasted 24–46 min, with an average of 33 min. Participants were between 23 years and 61 years old and had worked as a midwife for 10 months to 23 years (Table 2). Participants were employed in 8 out of 12 provinces in the Netherlands and were connected to 22 of the 71 MCCs. Most midwives worked fulltime and the estimated number of vulnerable pregnant women in their care ranged from 2 to 120 vulnerable women per year, based on their own definition of a vulnerable pregnant women.

### 3.2. Definition

Participants generally defined vulnerable women as women who experience psychosocial problems and, therefore, need extra care. Frequently indicated signs of vulnerability were low education, and financial, psychological, or housing problems. Some midwives suggested that a combination of those problems and a lack of a supporting social network or coping skills lead to vulnerability. Less commonly indicated signs included young maternal age, having a migration background, substance use, and not having a stable relationship (Table 3). Participants pointed out that there are various degrees of vulnerability.

### 3.3. Current Care

Approximately half of the participants used guidelines from the MCC to care for vulnerable women. Each MCC had their own policies for providing care. Some midwives provided more frequent consultations for vulnerable women and used vulnerability detection tools in the form of checklists or questionnaires. Midwives regularly contacted organizations and care providers such as youth health care, municipal community teams, psychologists, gynecologists, and social workers, for consultation or referral. Additionally, the advice and reporting center for domestic violence and child abuse, Safe at Home, was sometimes contacted for consultation. Some MCCs employed a coordinating care provider specialized in vulnerable pregnant women, who ensured that vulnerable women were referred to the right organization for their (psychosocial) problems.

### 3.4. TDF Domains

#### 3.4.1. Beliefs about Consequences

In general, midwives believed that the extra care offered to vulnerable pregnant women had positive consequences for those women. However, participants also indicated that sometimes too many organizations were involved in the care, resulting in confusion.

#1: “I think, because a lot has been set up and there are many projects, vulnerable pregnant women have a better chance. Because care has already been given in those first 1000 days [from conception onwards], they [the mothers] do not fall behind, so the chance of success in parenthood is much greater.”

#### 3.4.2. Professional Role and Identity

Midwives thought that providing psychosocial care for vulnerable pregnant women, in addition to medical care, was part of their professional role. They considered it their task to identify vulnerabilities and refer clients to relevant specialized care.

#1: “I think that, as a midwife, you are a coordinating care provider, you do not have to solve everything, but you do have to identify when extra care is needed. And the moment you notice this, you also actively look for the extra care that is available and how it can be provided.”

#### 3.4.3. Optimism and Emotion

Every participant experienced their work positively. Their work was generally described as varied. Some called their work demanding, mainly due to irregular shifts. When caring for vulnerable women, some midwives felt tense and uncertain. They indicated feelings of worry and compassion for the women’s problems. Additionally, midwives said caring for the vulnerable population required a lot of energy. Other midwives perceived care for vulnerable women as challenging and felt that it was extra important.

#8: “I think you feel more important. Especially because vulnerable pregnant women are simply much more in need than “normal” pregnant women.”

#### 3.4.4. Goals, Intentions, and Reinforcement

Midwives aimed to optimize the situation for mother and child, and to enable pregnant women to make a strong start to parenthood. #13: “That you actually help them [vulnerable women] in pregnancy. That she actually has enough tools to continue the pregnancy as healthy as possible and thus ultimately will be able to raise her baby.” Additionally, midwives wanted to make sure that the necessary care was on track. All midwives intended to provide extra care for vulnerable women to achieve these goals. Almost all participants were motivated by seeing improvements in the situation for the mother and the child and by gratitude from pregnant women.

#### 3.4.5. Skills and Knowledge

Most participants mentioned that knowing local relevant psychosocial organizations is necessary to care for vulnerable pregnant women.

#2: “I think you need to understand what the connections [between different care providers and organizations] are. … Whom you can involve.”

Skills reported to be necessary included communication skills, empathic ability, objective attitude, and intuition. Midwives indicated a lack of training regarding vulnerable women during the midwifery education.

#### 3.4.6. Environmental Context and Resources

Lack of time or financing were frequently mentioned barriers, often mentioned together. Some midwives only mentioned limited consultation time, while others mentioned the increased workload of caring for vulnerable women, without receiving additional financial compensation. Midwives indicated they sometimes received additional financing from health insurances for pregnant women who live in neighborhoods that are registered as being deprived, but that this was insufficient and did not apply for all deprived areas or all vulnerable women.

#12: “I think that current care pays too little to properly care for vulnerable pregnant women. There should actually be a kind of separate rate for that. So, not just from the rates of a deprived neighborhood, because we notice that some deprived areas where nobody has a job and they eat dry bread at the end of the month, are not indicated as a deprived neighborhood.”

In addition, the COVID-19 pandemic and ensuing regulations at the time of the interviews was a barrier. Use of face masks and intakes by telephone were seen as barriers by most midwives, resulting in missing signs of vulnerability.

Many participants mentioned working with a care coordinator, having a guideline for the care for vulnerable pregnant women, and working with a vulnerability detection tool as facilitators. All these facilitators were perceived as beneficial because they helped structure identifying and referring vulnerable women, and provided an overview of involved organizations and care providers.

#6: “A guideline helps a lot. I think that standardization of care and making agreements about it in the region is very good.”

#### 3.4.7. Social Influences

Participants indicated they found it easier to provide care for someone who was open to care, compared to someone who was not. However, they also thought that there was no difference between the actual care they provided to those women. Language barriers between care providers and pregnant women who do not speak English or Dutch were also mentioned to impede care.

Most participants indicated that colleagues within their midwifery practice mainly had a supportive and positive influence on the care provided.

#17: “Well, we help each other a lot. We consult very easily, and certainly about these types of cases [of vulnerable pregnant women] …. Like, “think about that”, or “how could we handle this?”.”

Participants further mentioned that other care providers (e.g., general practitioners, gynecologists, and youth health care) also influenced the care. Their influence was mostly positive because they contributed to extra psychosocial care. However, sometimes they had a negative influence on care, because they did not consider the pregnant woman as vulnerable, did not see the importance of providing extra psychosocial care, or because they had long waiting lists (e.g., psychologists). Good communication between colleagues and other care providers was crucial, ensuring that everyone was aware of which care was needed and which care was already provided. Midwives frequently mentioned that often too many organizations were involved, complicating collaboration. Consequently, midwives sometimes perceived a lack of clarity about the role of the different organizations involved.

#6: “Sometimes we [the midwives and other care providers] have a failure in our communication. Also, because we do not know each other that well, because there are so many organizations.”

#### 3.4.8. Beliefs about Capabilities

There were substantial differences in the self-efficacy reported by midwives regarding care for vulnerable women. However, there was no clear relationship between experience and self-efficacy. A midwife with 23 years of experience (#15) said: “I am not sure about the care [for vulnerable women] at all. No.”, while a midwife with 2 years of experience (#9) said: “Yes. I am quite sure about it [the care for vulnerable women]. And when I am uncertain what I should do with something, I discuss this with colleagues.” There was also no apparent relationship between experience with caring for vulnerable women (i.e., the number of vulnerable women in care) and self-efficacy regarding care for vulnerable women.

#### 3.4.9. Behavioral Regulation

Midwives often had a structure within the practice to get vulnerable women who missed their appointments back into care. The midwives sought contact via email, WhatsApp, telephone, or visited them in person. Midwives also checked whether the women had made a new appointment. This was experienced as helpful.

#### 3.4.10. Memory, Attention, and Decision Processes

Participants found that vulnerability detection tools and standardization of questions during the intake helped to identify vulnerabilities.

#3: “We do have an intake form, which they fill out before they come to the intake. That is just very nice, then you can get the signs [indicators for vulnerability] from there.”

## 4. Discussion

This study explored the perspectives, perceived barriers, and perceived facilitators of midwives in the Netherlands regarding current care for vulnerable pregnant women. Midwives operated differently, but all provided extra psychosocial care to vulnerable pregnant women when they considered it necessary. Generally, they expected positive consequences resulting from extra care. Moreover, all midwives considered it their task to identify and refer vulnerable women, and intended to improve the situation for mother and child. Midwives indicated multiple barriers and facilitators regarding care for vulnerable women. The main barriers were related to environmental context and resources (i.e., lack of time, insufficient financing) and social influence (i.e., uncooperative vulnerable women, too many organizations being involved, lack of communication between care providers). Main facilitators were also related to environmental context and resources (i.e., presence of guidelines) and social influence (i.e., care coordinators, cooperative vulnerable women, consultation with colleagues, and good communication between different care providers), but also to knowledge (i.e., about the local psychosocial organizations), skills (i.e., good communication skills), and memory/attention and decision processes (i.e., vulnerability detection tools).

For this study we chose not to provide a general definition of vulnerable women, but to explore the definitions used by midwives themselves. The definitions of vulnerable pregnant women given in the current study were similar, though not completely identical to the definition of Scheele et al. [5]. The definitions given by the midwives in the current study included indicators similar to the physical, psychological, cognitive, and/or social risk factors listed by Scheele et al. [5]. Social networks or coping skills were less often explicitly mentioned by current participants. This might also have consequences for the number of reported vulnerable women in the care of each participant, which ranged from 2 to 120 per year. This large range may be partly explained by differences in the reported definitions by participants on the one hand, and partly by actual differences in percentages of vulnerable women, on the other hand. These results should therefore be considered explorative.

In the current research, years of experience as a midwife and the number of vulnerable pregnant women in care did not seem to be related to the midwives’ self-efficacy. This is unexpected because mastery experience is often the most important source of self-efficacy [24]. Therefore, more experience with care for vulnerable pregnant women was expected to increase self-efficacy regarding this care. However, previous experiences might not always be mastery experiences. If perceived as non-successful, previous experiences might undermine midwives’ self-efficacy [24]. Additionally, self-efficacy not only depends on mastery experience, but also on vicarious experience, verbal persuasion, and emotional state [24]. Bedwell et al. [25] found that the principal factor affecting self-efficacy in maternity care was the influence of colleagues (including verbal persuasion and vicarious experience). This could also be true for self-efficacy in the care for vulnerable pregnant women. Another factor that may have influenced self-efficacy is that, in medical education, increasing emphasis is placed on developing soft skills like teamwork and communication [26]. Further research is needed to identify factors contributing to self-efficacy by midwives regarding care for vulnerable pregnant women.

The current research suggests that not all midwife-led care practices used vulnerability detection tools and that not all MCCs had local guidelines for caring for vulnerable pregnant women. Those that had, indicated that it facilitated identifying and referring vulnerable women, thereby increasing effectiveness of care. This lack of guidelines and vulnerability detection tools in some MCCs is consistent with previous research [10]. Vulnerability detection tools and guidelines can provide structure to prenatal risk management and have been shown to be feasible for use in Dutch maternity care [27,28]. Using such tools, a more organized screening approach can be employed, which is suggested to lower perceived burden for midwives [29]. To overcome the lack of tools and local guidelines, a national guideline could be developed by the Royal Dutch Association of Midwives. Similar screening tools were suggested to improve antenatal care in Belgium [30].

Additionally, communication between different organizations or care providers from the psychosocial (e.g., social workers) and medical domain (e.g., midwives) were found important. Midwives indicated that there are too many organizations involved, leading to lack of insight into local networks. This makes interdisciplinary communication more difficult. A good structure can help multidisciplinary collaboration [31]. Standardization of care could provide structure. This standardization could be provided by developing documents which outline local networks, including the different organizations and care providers that are involved in the care for vulnerable pregnant women and how to contact them. These documents could be developed by MCCs, as they are responsible for establishing multidisciplinary collaboration [19]. Moreover, multidisciplinary communication can be improved by increasing understanding of perspectives and competencies of other care providers [31]. This could be achieved by having midwives form connections between the psychosocial and medical domain.

Care coordinators for vulnerable pregnant women could further help in gaining an overview of involved organizations. This is in line with previous research which found that working in networks coordinated by care coordinators is associated with improved quality of care, increased patient satisfaction, and increased efficiency [32,33]. Therefore, working with care coordinators within a network is recommended. In previous research, midwives from midwife-led care practices revealed high scores in connectivity with other care providers in the network [34]. Therefore, it is assumed that they would be suitable as care coordinators in Dutch maternity care.

In the current research, a lack of time was also found to be a barrier. This is in accordance with the previous literature, where a high workload and limited consultation time were found to act as barriers for screening and referral of substance abuse [35]. The time and financial barriers found in the current study are intertwined. If midwife-led practices would receive additional financial compensation in proportion to the number of vulnerable pregnant women in their practice, then this could be used to pay for the increased time investment.

### 4.1. Strengths and Limitations

One of the strengths of the current research is the spread of participating midwives across the Netherlands. Midwives from 8 of the 12 provinces and 31% of the MCCs were included in the study. There is also a variety in age and years of experience of the participants. These sample characteristics increase the generalizability of the study in the Netherlands. Another strength is that the codebook was validated, showing a strong interrater reliability. Additionally, data saturation was reached in the current sample of 19 participants. Previous research showed that saturation can be achieved within a smaller sample size (N = 9–17) [36]. The assumption in the current study was that sufficiently data saturation was reached when two consecutive interviews did not result in any new information. This saturation occurred after 19 interviews, as both the 18th and 19th interview did not add any new information or codes. In line with this, previous qualitative TDF-based research contains similar or smaller sample sizes (N = 11–16) [18,37]. Nonetheless, it would be valuable to examine generalizability of the current findings in a large-scale quantitative study.

The current research also has several limitations. First, the definitions of vulnerability given by the midwives differed slightly from this research’s formal definitions [5]. As a result, midwives may have had different women in mind when talking about vulnerable women. It might also have influenced the number of vulnerable women they reported to care for per year. Second, during recruitment for potential participants, midwives that already felt closely involved in care for vulnerable pregnant women might have been more willing to participate. Thus, selection bias may have occurred. This might explain the high observed motivation of participants to give extra psychosocial care to vulnerable women. However, it could also be the case that some of the participating midwives were not aware of the fact that they were not providing adequate care, and their reflections were overly positive. Third, the COVID-19 pandemic could have influenced results. The cited barriers and facilitators may only be applicable during the current COVID-19 situation or may have influenced other barriers and facilitators. This may reduce the generalizability of the results.

### 4.2. Recommendations

In summary, development of a national guideline and local overviews of involved organizations, can support midwives and increase effectiveness of care for vulnerable pregnant women. Guidelines should recommend the use of vulnerability detection tools and care coordinators. Local overviews of relevant care providers should include clear instructions on when to refer to them and how to contact them. Furthermore, midwives from midwife-led care practices can establish connections between the psychosocial and medical domain and could act as care coordinators in the care for vulnerable pregnant women. Moreover, the possibility of financing extra care for vulnerable pregnant women should be explored.

## 5. Conclusions

The current study provided insight into the perspectives, perceived barriers, and perceived facilitators of midwives in the care for vulnerable pregnant women. The findings suggest that midwives are highly motivated to care for vulnerable women. However, they experience multiple barriers in this care. A national guideline, local overviews of involved organizations, and proactive midwives who ensure connections between the psychosocial and medical domain could help to overcome these barriers, and therefore, improve care for vulnerable pregnant women and achieve health benefits for mothers and children.

## Figures and Tables

**Table 1:** Example questions of the interview guide including all domains of the Theoretical Domains Framework and background.

Domains	Example Question
Background	Where do you work in The Netherlands?
Optimism	How do you experience your job as a midwife in general?
Memory-attention-decision-processes	How many vulnerable pregnant women do you care for per year?
Goals	What goal do you envision when caring for vulnerable pregnant women?
Intentions	As a midwife, are you willing to provide extra care for vulnerable pregnant women?
Knowledge	What knowledge do you need as a midwife to care for vulnerable pregnant women?
Skills	What skills do you need as a midwife to care for vulnerable pregnant women?
Professional role and identity	What do you think is your role as a midwife in the care for vulnerable pregnant women?
Beliefs about capabilities	How confident are you of your care for vulnerable pregnant women? ^1^
Beliefs about consequences	What do you think are the consequences of current care for vulnerable pregnant women? ^1^
Reinforcement	What motivates you in the care for vulnerable pregnant women?
Environmental context/resources	What are the challenges in the care for vulnerable pregnant women? ^1^
Social influences	Does the vulnerable pregnant woman influence you in the care you provide to her? If so, how?
Emotion	How do you feel when caring for a vulnerable pregnant woman?
Behavioral regulation	Are there ways of working that encourage you to provide extra care for vulnerable pregnant women? ^1^

^1^ Regarding to identifying vulnerable women, referring vulnerable women to specialized care providers, and increasing the attendance of vulnerable women in prenatal care.

**Table 2:** Characteristics of the 19 participants from midwife-led care practices.

Participant	Age (Years)	Completed Higher Education Other than Midwifery	Time Working as a Midwife Rounded to Years	Working Hours (Fulltime/Parttime)	Estimated Number of Vulnerable Pregnant Women in Their Care (Women per Year)
#1	24	No	3	Fulltime	20–25
#2	28	Yes ^1^	3	Fulltime	60–120
#3	26	No	3	Fulltime	10
#4	30	No	4	Fulltime	35–70
#5	24	No	3	Fulltime	36–60
#6	28	No	2	Fulltime	4
#7	57	Yes ^2^	12	Fulltime	20–24
#8	24	No	2	Fulltime	24–36
#9	34	No	2	Fulltime	10–15
#10	41	No	18	Parttime	110–115
#11	24	No	2	Fulltime	2–3
#12	42	No	19	Fulltime	60–120
#13	35	No	11	Fulltime	10
#14	23	No	2	Fulltime	10
#15	61	No	23	Parttime	15–16
#16	25	No	1	Fulltime	5–10
#17	33	No	12	Fulltime	60–120
#18	42	No	16	Fulltime	100
#19	28	No	6	Fulltime	60–120

^1^ Bachelor of applied science in pedagogy. ^2^ Bachelor of applied science in nursing.

**Table 3:** Number of midwives (N = 19) that indicated the specified signs of vulnerability in their definition of vulnerable pregnant women.

Signs of Vulnerability	Number of Midwives Who Included the Sign in Their Definition *n* (%)
Financial problems	14 (74)
Low level of education	13 (68)
Psychological problems	12 (63)
Bad living conditions/housing problems	11 (59)
Young maternal age	7 (37)
No social support and/or coping skills	6 (32)
Migration background	6 (32)
Substance use	6 (32)
Not having a stable relationship	5 (26)

## Data Availability

Data supporting the findings of this study are available from the corresponding author on request.

## Appendix A. Interview Guide

Thank you for participating in this interview and in my research. My name is [name interviewer] and I am a midwife and master student at Maastricht University. The purpose of this research is to map the experiences of midwives regarding the current care for vulnerable pregnant women in the Netherlands to gain insight in the factors that make this care easier or harder. All questions are regarding your own perspectives, experiences, and feelings about the care for vulnerable pregnant women. There are no right or wrong answers. This interview will take about 30–60 min. You signed the form of consent and with that, you agreed to be audio recorded, you indicate that you are voluntarily participating in the research, and you know that your data will be processed anonymously. Is that right? [wait for answer].

Do you have any questions before we start? [wait for an answer]. OK, then I will start the recording.

**Table A1:** Interview guide.

**Introduction**	Today, it is … (date) …. The goal of the research is to map the experiences of midwives regarding the current care for vulnerable pregnant women in the Netherlands, to explore which factors can be experienced as helpful or as a barrier.
**Theoretical Domains**	**Questions**
Background	Where do you work in the Netherlands? How old are you? How long have you been working as a midwife? How many hours do you work as a midwife per week (fulltime, part-time)? Did you finish another higher education, other than midwifery? If so, which? Which MCC are you connected with?
Optimism	How do you experience your job as a midwife in general?
Memory, attention, and decision processes	What do you understand as a vulnerable pregnant woman? How many vulnerable pregnant women do you care for per year? How is the current care surrounding vulnerable pregnant women handled in the MCC? ^1^
Goals	What goal do you envision when caring for vulnerable pregnant women?
Intentions	As a midwife, are you willing to provide extra care for vulnerable pregnant women?
Social and professional role and identity	What do you think is your role as a midwife in the care for vulnerable pregnant women? And what is the responsibility of pregnant women themselves? And what of a different care provider? Why?
Knowledge and skills	What knowledge and skills do you need as a midwife to care for vulnerable pregnant women? ^1^ Do you have that knowledge and those skills? If not: what is missing? And what is needed to obtain this knowledge/skill.
Beliefs about capabilities	How confident are you of your care for vulnerable pregnant women? ^1^ Where are you most confident in: identifying, referring, or increasing the presence at prenatal check-ups. Why? Is there anything you are particularly unsure about?
Beliefs about consequences	What do you think are the consequences of current care for vulnerable pregnant women? ^1^ What are the advantages and disadvantages?
Environmental context and resources	What are the challenges in the care for vulnerable pregnant women? ^1^ What helps you in caring for vulnerable pregnant women? ^1^ Do you have enough resources to care for vulnerable pregnant women? (time, pathways, support, agreements with other healthcare providers, communication skills, etc.) Do you have guidelines for the care for vulnerable pregnant women within the MCC or within the practice? To what extent does COVID-19 play a role in the care for vulnerable pregnant women?
Reinforcement	What motivates you in the care for vulnerable pregnant women? (money, better children’s health, happy parents, etc.)
Social influences	Does the vulnerable pregnant woman influence you in the care you provide to her? If so: how does she affect it? Which people influence the care you provide to vulnerable pregnant women? (family, gynecologists, MCCs, regional partnerships) How do these people influence it? What do you think your colleagues within the MCC think about the care for vulnerable pregnant women? Do you talk about this?
Behavioral regulation	Are there ways of working that encourage you to provide extra care for vulnerable pregnant women? ^1^
Emotion	How do you feel when caring for a vulnerable pregnant woman? Do you ever avoid raising the topics of identifying and referral of care for vulnerable pregnant women because it makes you feel a certain way?
All questions of the interview are now answered. Is there anything you would like to add or ask? [wait for an answer] Thank you for participating in this research. You really helped me by describing your perspectives, experiences, and feelings regarding the current care for vulnerable pregnant women. You can always email me if you have any question about the interview or the research. I will stop the recording now.	

^1^ Regarding to identifying vulnerable women, referring vulnerable women to specialized care providers, and increasing the attendance of vulnerable women in prenatal care. Abbreviations: MCC, Maternity Care Collaboration.

## Appendix B. Codebook

**Table A2:** Codebook.

Main Nodes	Secondary Nodes	Tertiary Nodes	Definition
Background			The circumstances or situations prevailing at the time of the interview.
	Age		The length of time that the participant has lived.
	Education		The higher education completed by the participant other than midwifery.
	Work		Remarks made by the participant regarding work.
		Number of vulnerable pregnant women	The number of vulnerable pregnant women cared for by the participant in the past year.
		Time working as a midwife	How many years the participant has been working for as a midwife.
		Working hours	The participants’ average working hours per week.
		Working place	General area where the participant is working at the moment of the interview.
	MCC		The Maternity Care Collaboration to which the participant is connected.
Attitude, optimism, and emotion			A feeling or opinion about something.
	Emotions or ideas about work in general		A strong feeling deriving from one’s circumstances, mood, or relationships with others or ideas about work in general.
	Emotions when caring for a vulnerable pregnant woman		A strong feeling deriving from one’s circumstances, mood, or relationships with others when caring for a vulnerable pregnant woman.
Behavioral regulation			Behavior of the participant that influence the care for vulnerable pregnant women or the behavior of the pregnant woman regarding that care.
Beliefs about capabilities			Confidence the participant has in their abilities to care for vulnerable pregnant women.
Beliefs about consequences			Benefits or drawbacks of the current care for vulnerable pregnant women.
	Advantages of current care		Benefits of the current care for vulnerable pregnant women.
	Dis-advantages of current care		Drawbacks of the current care for vulnerable pregnant women.
Current care			Remarks made by the participant regarding the current care provided for vulnerable pregnant women.
	Current guidelines		The current guidelines for caring for vulnerable pregnant women (not) used by the participant.
	Others		Other remarks made by the participant regarding the current care provided for vulnerable pregnant women.
Environmental context and resources			Any circumstances of the participants’ situation or environment.
	Barriers		Circumstances or obstacles that prevent the participants in providing adequate care for vulnerable pregnant women.
		COVID-19	Anything related to COVID-19 that prevents the participants in providing adequate care for vulnerable pregnant women.
		Finance	Anything related to financing that prevents the participants in providing adequate care for vulnerable pregnant women.
		Time	Anything related to a lack of time that prevents the participants in providing adequate care for vulnerable pregnant women.
		Too much organizations	Anything related to an excess of organizations that prevents the participants in providing adequate care for vulnerable pregnant women.
		Others	Anything that prevents the participants in providing adequate care for vulnerable pregnant women that does not fall under any of the beforementioned categories.
	Facilitators		Circumstances that aid the participants in providing adequate care for vulnerable pregnant women.
		Care coordinator	Anything related to the presence of a care coordinator that aids the participant in providing adequate care for vulnerable pregnant women.
		Guideline	Anything related to the presence of guideline(s) that aid the participants in providing adequate care for vulnerable pregnant women.
		Consultation	Anything related to consultation of colleagues that aid the participants in providing adequate care for vulnerable pregnant women.
		Others	Anything that aids the participants in providing adequate care for vulnerable pregnant women that does not fall under any of the beforementioned categories.
	Neutral		Any circumstances of the participants’ situation or environment that is neither a barrier nor a facilitator.
		COVID-19	Any circumstances of the participants’ situation or environment related to COVID-19 that are neither barriers nor facilitators.
		Others	Any circumstances of the participants’ situation or environment that are neither barriers nor facilitators and does not relate to COVID-19.
Goals			Mental representations of outcomes that the participants aim for when caring for vulnerable pregnant women.
Intentions			Mental representations of the behaviors that the participants aim for when caring for vulnerable pregnant women.
Knowledge			The awareness of the existence of facts or information.
	Necessary knowledge		Knowledge that is necessary to care for vulnerable pregnant women.
		Network	Any knowledge related to the network of organizations that is necessary to refer vulnerable pregnant women.
		Others	Any knowledge that is necessary to care for vulnerable pregnant women and is not related to the network of the participants.
	Definitional knowledge		Knowledge about the definition of vulnerable pregnant women.
Reinforcement			Anything that motivates the participants to care for vulnerable pregnant women.
	Gratitude for care		Anything related to the gratitude vulnerable pregnant women give the participants that motivates the participants to care for vulnerable pregnant women.
	Improvement in situation mother and child		Anything related to improvements in the situation of vulnerable pregnant women and/or their child that motivates the participants to care for vulnerable pregnant women.
	Others		Anything that motivates the participants to care for vulnerable pregnant women that does not fall under any of the beforementioned categories.
Skills			Abilities or proficiencies related to caring for vulnerable pregnant women.
	Communication skills		Communication skills related to caring for vulnerable pregnant women.
	Empathy		Empathic capabilities related to caring for vulnerable pregnant women.
	Being objective		The skill of being objective related to caring for vulnerable pregnant women.
	Intuition		Intuition related to caring for vulnerable pregnant women.
	Others		Abilities or proficiencies related to caring for vulnerable pregnant women that do not fall under any of the beforementioned categories.
Social influences and norms			Interpersonal processes that can cause participants to change their thoughts, feelings or behaviors when caring for vulnerable pregnant women.
	Pregnant women		Interpersonal processes between pregnant women and the participant that can cause participants to change their thoughts, feelings or behaviors when caring for vulnerable pregnant women.
		Language	Anything related to language barriers that prevents the participants in providing adequate care for vulnerable pregnant women.
	Colleagues		Interpersonal processes between colleagues and the participant that can cause participants to change their thoughts, feelings or behaviors when caring for vulnerable pregnant women.
		Communication barrier	Anything related to communication between care providers that prevents the participants in providing adequate care for vulnerable pregnant women.
		Communication facilitator	Anything related to communication between care providers that aid the participants in providing adequate care for vulnerable pregnant women.
	Others		Interpersonal processes that can cause participants to change their thoughts, feelings or behaviors when caring for vulnerable pregnant women that do not fall under any of the beforementioned categories.
Professional role and identity			The role that the participants have in the care for vulnerable pregnant women.
Memory, attention and decision processes			The ability of the participants to remember, focus selectively on aspects of the environment and choose between two or more alternatives in relation to the care for vulnerable pregnant women.
